# Critical windows: timing of child maltreatment exposure and adult mental health

**DOI:** 10.1186/s13034-026-01188-0

**Published:** 2026-10-06

**Authors:** Daniëlle Otten, Inga Schalinski, Jörg M. Fegert, Andreas Jud, Elmar Brähler, Vera Clemens

**Affiliations:** 1 Department of Child and Adolescent Psychiatry, Psychosomatics and Psychotherapy, University Hospital Ulm, Steinhövelstr. 5, 89075 Ulm, Germany; 2 German Center for Mental Health (DZPG), Partner Site Ulm, Ulm, Germany; 3 German Center for Child and Adolescent Health (DZKJ), Partner Site Ulm, Ulm, Germany; 4 Department of Human Sciences, Institute of Psychology, University of the Bundeswehr Munich, Werner-Heisenberg-Weg 39, D-85577 Neubiberg, Germany; 5 School of Social Work, ZHAW University of Applied Sciences Zürich, Pfingstweidstrasse 96, Zürich, 8005 Switzerland; 6 Clinic and Polyclinic for Psychosomatic Medicine and Psychotherapy, Rostock University Medical Center, Schillingallee 35, 18057 Rostock, Germany; 7 Department of Psychosomatic Medicine and Psychotherapy, University Medical Center of the Johannes Gutenberg-University Mainz, Untere Zahlbacher Straße 8, 55131 Mainz, Germany

## Abstract

**Background:**

Despite being recognized as the leading risk factor for mental disorders, child maltreatment (CM) remains insufficiently understood regarding type and timing of exposure. This study examines which developmental periods of exposure to CM subtypes are most strongly associated with adult mental disorders in women and men.

**Methods:**

Data from a German survey study from 2021 was used. The sample included *N* = 1,278 women [51.8%] (*M*_age_ = 50.3; SD = 17.6) and *N* = 1,188 men [48.2%] (*M*_age_ = 51.1; SD = 17.6). CM was assessed using the ISPCAN Child Abuse Screening Tools Retrospective version questionnaire. Conditioned Random Forest regression analyses were applied to examine the relevance of type and timing of CM for internalising [IMD] and externalising mental disorders [EMD] in adulthood. SHapley Additive exPlanations analyses were performed as post-hoc tests.

**Results:**

Timing of exposure to emotional abuse was most strongly associated with IMD; for women between ages 10–16 (variable importance [VI] ranging from 0.255 to 1.014), and for men ages 13 and 14 (VI: 0.349 and VI: 0.504). For men, EMD were most closely linked to emotional abuse between ages 10–16 (VI: 0.174–0.968), for women to exposure to sexual abuse between ages 13–15 (VI: 0.136–0.331) and neglect between ages 11–17 (VI: 0.098–0.331).

**Conclusion:**

Distinct developmental windows of vulnerability to varying subtypes of CM shape adult mental health differently in women and men. Considering timing of subtypes of CM may enhance diagnosis and prevention strategies aimed at reducing lifetime mental health risks.

**Supplementary Information:**

The online version contains supplementary material available at https://doi.org/10.1186/s13034-026-01188-0.

## Introduction

 Child maltreatment (CM) refers to *all types of abuse and neglect of a child under the age of 18 by a parent*,* caregiver*,* or another person in a custodial role that results in harm*,* the potential for harm*,* or threat of harm to a child* [1]. In Germany, 23.1% of all adults report having experienced at least one subtype of CM in childhood [2]. CM substantially contributes to child mortality and morbidity [3] and can have devastating consequences for a person’s mental and physical health in adulthood [4, 5].

Individuals exposed to CM are at increased risk for a broad range of mental disorders. Emotional neglect and emotional, sexual, and physical abuse are for example associated with depression, anxiety, substance abuse, personality disorders, eating disorders, and suicidal ideation or attempts in adulthood [6]. High levels of psychiatric comorbidity, i.e., co-occurrence of two or more individual mental disorders, exist. Support for the hierarchical internalising-externalising model was found [7] implying that broad categories of internalising mental disorders (IMD) and externalising mental disorders (EMD) exist (based on core symptoms that are typically expressed) and that each latent factor comprises several individual mental disorders. Exposure to CM increases the risk of both IMD (e.g., mood- and anxiety disorders or stress-related disorders) and EMD (e.g., disruptive behavior disoders or substande use) [4, 8, 9]. Furthermore, individuals with CM exposure show ealier symptom onset, higher symptom severity and comorbidty, and poorer treatment response compared to individuals without CM exposure, but with the same mental health diagnosis [10, 11].

Many studies do not account for the age at, or duration of, exposure of CM. Given the unique and complex heterogeneity of CM, the nature of the CM and the timing of exposure influence a child’s brain development causing lasting structural and functional neurobiological alternations [12]. CM for example influences the development of the hypothalamic-pituitary-adrenal (HPA)-axis [13, 14], the primary site for regulating the body’s stress response through releasing glucocorticoid hormones, which has a negative impact on numerous (mental) health outcomes [15, 16]. It alternates the body’s stress regulation system and diverts energy away from maintenance, growth, and repair processes (i.e., the allostatic load model). This could in turn lead to low-grade inflammation and immune system dysregulation [17]. Neurobiological alterations are therefore directly and indirectly associated with an increased risk for mental disorders [17, 18]). Focussing on a single subtype of CM or aggregating distinct maltreatment types into a risk score implicitly assumes that different experiences exert on development and health across all years of exposure in the same way, which is unlikely.

The frequency of maltreatment, its duration (i.e., experiencing CM over a longer period of time) and its timing (exposure during different ages and development periods) were found to be predictive of several neurobiological [19, 20] and mental health symptoms and disorders [21]. With regard to type and timing, studies in youth school samples revealed abuse at age five to be associated with IMD, whereas abuse at age 10 was associated with EMD [22]. In a study on out-of-home-placed youth, parental abuse at age six was predictive of internalising disorders, sexual abuse in late childhood predictive of internalising- and externalising disorders as well as peer violence in puberty [23]. Studies in transdiagnostic adult psychiatric populations have shown that physical neglect at age 5 and emotional neglect at ages 4–5 were linked to increased dissociative symptoms, whereas emotional neglect at ages 8–9 was associated with heightened depressive symptoms [24, 25], sexual abuse before the age of 5 and physical abuse after the age of 13 posing particularly high risks for adult depression and PTSD symptoms [26]. A previous meta-analysis could not yet identify consistent sensitive periods of CM for psychiatric outcomes and suggested considering timing of subtypes of CM [27]. Together, these findings underscore the importance of considering both the type and timing of CM exposure, as specific subtypes of maltreatment during development appear to differentially shape long-term mental disorders.

Differences between women and men in (strength of) associations between subtypes of CM or multiplicity (experiencing more than one subtype of CM) and mental disorders exist [28]. Only few studies report on importance of subtype and timing for mental health in women and men. Khan et al. found non-verbal emotional abuse at age 14 and emotional neglect at age 12 to be important for major depressive disorder in early adulthood for men, whereas for women peer emotional abuse at age 14 was most important [29].

This study aims to determine how type and timing of CM and global CM indicators (i.e., multiplicity, duration, and frequency) predict adult IMD and EMD in women and men.

## Methods

### Sample

A representative sample of the German population in 2021 was obtained by an opinion research institute (USUMA, Berlin). The country was systematically divided into regional areas and further categorized into 128 “networks” serving as sampling frame. Each network contained 258 sample points, proportionally reflecting the distribution of private German households, in which households were randomly selected. The Kish-Grid method was applied to ensure random participation. Included were individuals aged 16 years and older with sufficient understanding of the German language. Participants were given information about the study and provided informed consent. Face-to-face interviews and written self-report questionnaires were used to assess demographic characteristics and information on health.

The study was conducted in accordance with the Declaration of Helsinki and complied with the ethical guidelines of the International Code of Marketing and Social Research Practice of the International Chamber of Commerce and of the European Society of Opinion and Marketing Research. The study was approved by the Ethics Committee of the Medical Department of the University of Leipzig. Items of the AAPOR’s Code of Professional Ethics and Practices are reported in this section.

In total *N* = 2,515 individuals participated in this study equalizing a response rate of 42.6%. Main reasons for non-participation were refusal of the selected household or target person to participate as well as failure to contact a household after four attempts. This study included individuals identifying as either women or men aged 18 and older, which lead to a final study sample of *N* = 2,466 participants with an average age of *M* = 50.7 (SD = 17.6) including *N* = 1,278 women [51.8%] (*M*_age_ = 50.3; SD = 17.6) and *N* = 1,188 men [48.2%] (*M*_age_ = 51.1; SD = 17.6).

### Measures

#### Child maltreatment

CM was measured with the ISPCAN Child Abuse Screening Tools Retrospective-version questionnaire [30], developed for young adults and assessing CM all-encompassing. This tool retrospectively screened for neglect, physical-, emotional-, and sexual abuse before the age of 18 and included follow-up questions on age at time of exposure, frequency, severity and perpetrator. This screening instrument revealed adequate to good internal consistency and very good psychometric properties [31].

For each subtype of CM multiple acts were described. Affirmation of at least one act was defined as experience of the corresponding CM subtype. For each act described, study participants could additionally indicate at what age(s) exposure had taken place. Binary variables for each age-subtype combination were calculated.

A continuous variable for multiplicity (i.e., exposure to multiple subtypes of CM) was calculated by summing the different subtypes leading to a multiplicity score ranging from 0 to 4. Duration was defined as the total number of years of experienced CM (across subtypes) calculated with a sumscore ranging from 0 to 18. Frequency was allocated using the maximum answer score of the follow-up question “How often did this happen to you” with the answer categories: <10 times (coded 1), between 10 and 50 times (coded 2) and too many times to count (coded 3).

#### Internalising and externalising mental disorders

Individuals were asked if they received a diagnosis from a physician or psychotherapist for the following mental disorder: depression, anxiety, burn-out, post-traumatic stress disorder, borderline and other personality disorder, eating disorder, attention deficit disorder (hyperactivity), schizophrenia, alcohol- and/or drug addiction. Based on the Hierarchical Taxonomy of Psychopathology model (using codes from the International Classification of Diseases, 10th Revision) depression, anxiety, burn-out, posttraumatic stress disorder, and eating disorders were combined in a sumscore ranging from 0 to 5 indicating IMD. EMD included personality disorders, attention deficit disorder, schizophrenia, and addiction disorders (sumscore ranging from 0 to 4).

#### Covariates

Current ageof the respondents, migration background, partnership and monthly equivalised net household income in euros were used as covariates as they are known to correlate with mental disorders.

### Statistical analyses

The characteristics of CM were compared between women and men using χ2 tests for categorical variables and t-test for continuous variables.

Conditioned random forest regression (cRF), a machine-learning based regression technique that uses decision trees as the base learners [32], was applied to test the predictive strength of CM characteristics on IMD and EMD in adulthood. cRF creates a forest of multiple decision trees that differ from each other as they are each generated from a different subset of the data and its number of predictors can be considered at each decision point. New data are run through this forest of trees followed by averaging the outcomes of all trees. By randomly permuting each variable in turn and determining how much this degrades the model fit, the importance of each predictor variable is estimated. A higher and significant variable importance (VI) score implies that a specific variable is more predictive of IMD or EMD than the other variables included in the model. In order to statistically validate the VI scores, cRF was performed 5,000 times, each time reshuffling the model variables to simulate random chance and recalculating VI for each predictor. This technique has a high prediction accuracy, is unsusceptible to multicollinearity, and does not require variables to be normally distributed. The ‘party’ package in R was used for these analyses [33].

Type and timing, multiplicity, duration, and frequency as well as age and household equivalised income were included in the models. Due to the impaired understanding and remembrance of events happening in early childhood, timing variables until the age of three were excluded from the analyses. Competitive hypothesis testing was used to identify the strongest predictors. Timing variables of subtype exposure were tested against the predictive strength of the global scores. All analyses were stratified for sex.

To enhance the interpretability of our conditional random forest models and examine the direction and strength of our timing variables for each subtype exposure, we applied SHapley Additive exPlanations (SHAP) analyses [34]. SHAP values quantify each predictor’s contribution to individual predictions by computing the average marginal effect across all possible feature coalitions. SHAP values were derived from ranger-based random forest models [35] fitted separately for men and women and estimated based on the full sample using the same predictor set as the main cRF model. Positive SHAP values imply that a variable increases the prediction, while negative values decrease it; the magnitude reflects the strength of the feature. SHAP based beeswarm plots visualized the individual predictions of subtype and timing of CM exposure for internalizing and externalizing mental disorders in women and men. These plots were restricted to individuals with CM exposure to improve interpretability by avoiding a dense cluster of near-zero values. The cRF and SHAP analyses were used to characterize multivariable association patterns and relative variable importance, not for causal effect estimation or predictive model deployment.

## Results

### Descriptive statistics

Women reported significantly more IMD than men [216 (17.1%) versus 118 (10.1%), *p* < 0.001]. Mean values of IMD were 0.32 ± 0.81 for women versus 0.17 ± 0.56 for men. EMD were significantly more often reported by men than by women [100 (8.5%) versus 56 (4.4%), *p* < 0.001], with mean values of 0.09 ± 0.32 for men versus 0.05 ± 0.25 for women. More women than men reported IMD and EMD [227 (18.1%) versus 175 (15.1%), *p* = 0.028] (see Table 1 and Supplementary Table 1).

**Table 1:** Sample characteristics stratified for women and men

	Women (*N* = 1,278)		Men (*N* = 1,188)		Significance (*p*-value)	
Internalising disorders (M ± SD)	0.32 ± 0.81		0.17 ± 0.56		< 0.001	
Externalising disorders (M ± SD)	0.05 ± 0.25		0.09 ± 0.32		< 0.001	
Timing neglect (N, %)						
Age 4	16 (1.3%)		20 (1.7%)		0.404	
Age 5	26 (2.0%)		31 (2.6%)		0.351	
Age 6	42 (3.3%)		58 (4.9%)		0.052	
Age 7	51 (4.0%)		64 (5.4%)		0.105	
Age 8	63 (4.9%)		77 (6.5%)		0.099	
Age 9	67 (5.2%)		78 (6.6%)		0.171	
Age 10	78 (6.1%)		78 (6.6%)		0.679	
Age 11	56 (4.4%)		52 (4.4%)		1.000	
Age 12	62 (4.9%)		62 (5.2%)		0.713	
Age 13	43 (3.4%)		46 (3.9%)		0.518	
Age 14	34 (2.7%)		37 (3.1%)		0.548	
Age 15	21 (1.6%)		33 (2.8%)		0.073	
Age 16	17 (1.3%)		22 (1.9%)		0.334	
Age 17	13 (1.0%)		20 (1.7%)		0.164	
Age 18	4 (0.3%)		13 (1.1%)		0.026	
Timing physical abuse (N, %)						
Age 4	17 (1.3%)		11 (0.9%)		0.447	
Age 5	37 (2.9%)		23 (1.9%)		0.150	
Age 6	54 (4.2%)		50 (4.2%)		1.000	
Age 7	72 (5.6%)		68 (5.7%)		0.931	
Age 8	90 (7.0%)		104 (8.8%)		0.117	
Age 9	92 (7.2%)		107 (9.0%)		0.104	
Age 10	109 (8.5%)		129 (10.9%)		0.056	
Age 11	98 (7.7%)		109 (9.2%)		0.191	
Age 12	86 (6.7%)		130 (10.9%)		< 0.001	
Age 13	67 (5.2%)		101 (8.5%)		0.001	
Age 14	67 (5.2%)		75 (6.3%)		0.282	
Age 15	48 (3.8%)		697(5.6%)		0.028	
Age 16	35 (2.7%)		40 (3.4%)		0.412	
Age 17	21 (1.6%)		22 (1.9%)		0.759	
Age 18	13 (1.0%)		13 (1.1%)		1.000	
Timing emotional abuse (N, %)						
Age 4	13 (1.0%)		6 (0.5%)		0.171	
Age 5	18 (1.4%)		14 (1.2%)		0.723	
Age 6	35 (2.7%)		30 (2.5%)		0.802	
Age 7	48 (3.8%)		44 (3.7%)		1.000	
Age 8	61 (4.8%)		65 (5.5%)		0.464	
Age 9	69 (5.4%)		69 (5.8%)		0.662	
Age 10	104 (8.1%)		93 (7.8%)		0.824	
Age 11	105 (8.2%)		105 (8.8%)		0.613	
Age 12	126 (9.9%)		118 (9.9%)		1.000	
Age 13	134 (10.5%)		110 (9.3%)		0.312	
Age 14	124 (9.7%)		112 (9.4%)		0.837	
Age 15	122 (9.5%)		109 (9.2%)		0.782	
Age 16	110 (8.6%)		105 (8.8%)		0.886	
Age 17	85 (6.7%)		66 (5.6%)		0.275	
Age 18	56 (4.4%)		42 (3.5%)		0.303	
Timing sexual abuse (N, %)						
Age 4	4 (0.3%)		2 (0.2%)		0.4688	
Age 5	3 (0.2%)		3 (0.3%)		1.000	
Age 6	7 (0.5%)		4 (0.3%)		0.551	
Age 7	5 (0.4%)		2 (0.2%)		0.455	
Age 8	4 (0.3%)		2 (0.2%)		0.688	
Age 9	6 (0.5%)		5 (0.4%)		1.000	
Age 10	9 (0.7%)		9 (0.8%)		1.000	
Age 11	17 (1.3%)		13 (1.1%)		0.714	
Age 12	27 (2.1%)		16 (1.3%)		0.167	
Age 13	36 (2.8%)		20 (1.7%)		0.078	
Age 14	48 (3.8%)		20 (1.7%)		0.002	
Age 15	51 (4.0%)		16 (1.3%)		< 0.001	
Age 16	63 (4.9%)		12 (1.0%)		< 0.001	
Age 17	57 (4.5%)		10 (0.8%)		< 0.001	
Age 18	32 (2.5%)		20 (1.7%)		0.164	
Global scores CM (M ± SD)						
Multiplicity (i.e., sumscore subtypes CM)	0.69 ± 1.09		0.71 ± 1.08		0.712	
Duration (i.e., sumscore years experienced CM)	1.71 ± 3.30		1.81 ± 3.42		0.446	
Frequency CM	0.56 ± 0.91		0.57 ± 0.92		0.763	
Confounders						
Age at survey participation	50.6 ± 17.9		49.5 ± 18.2		0.136	
Migration background	189 (15.4%)		166 (14.4%)		0.258	
Partnership (living together with partner)	641 (50.7%)		675 (57.4%)		< 0.001	
Net household equivalised income (Euros)	1934 ± 940		2103 ± 1076		< 0.001	

*CM* child maltreatment

### Internalising and externalising mental disorders

#### Importance of timing of subtypes child maltreatment

**Fig. 1:**
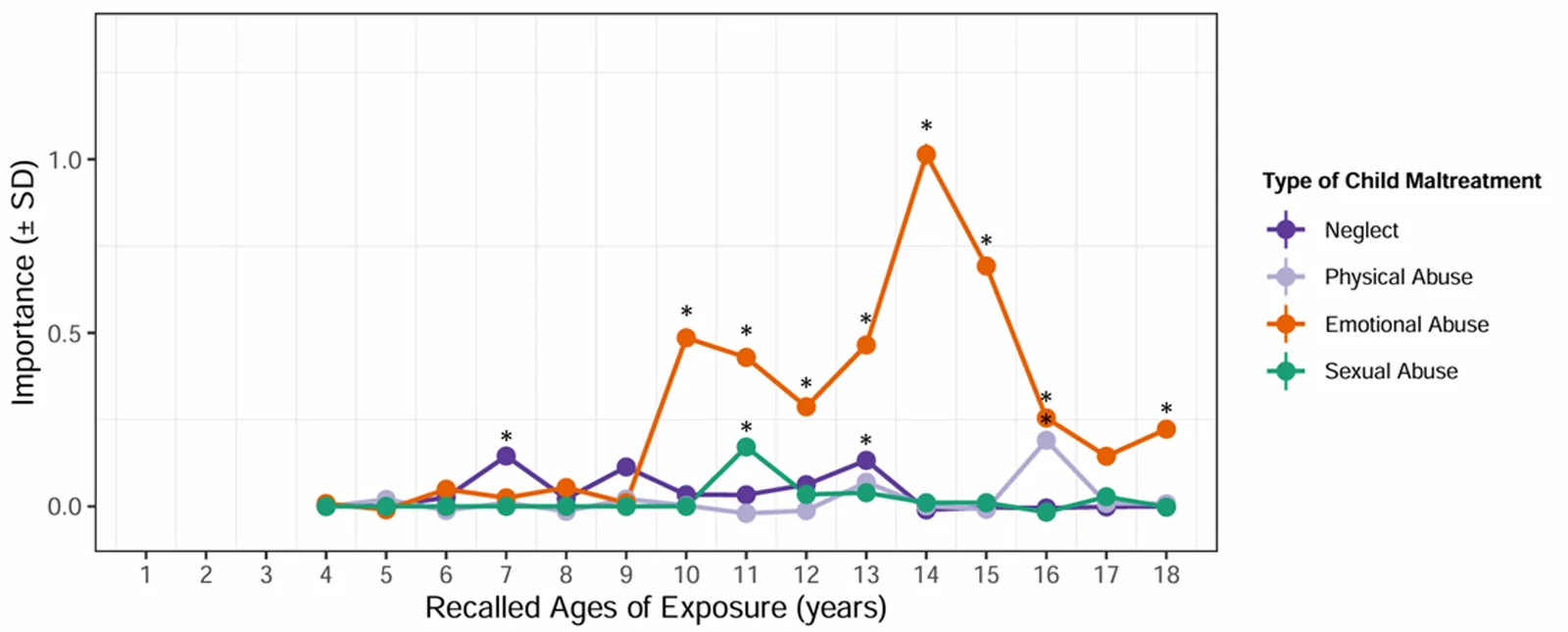
Importance of child maltreatment for adult internalising disorders in women

**Fig. 2:**
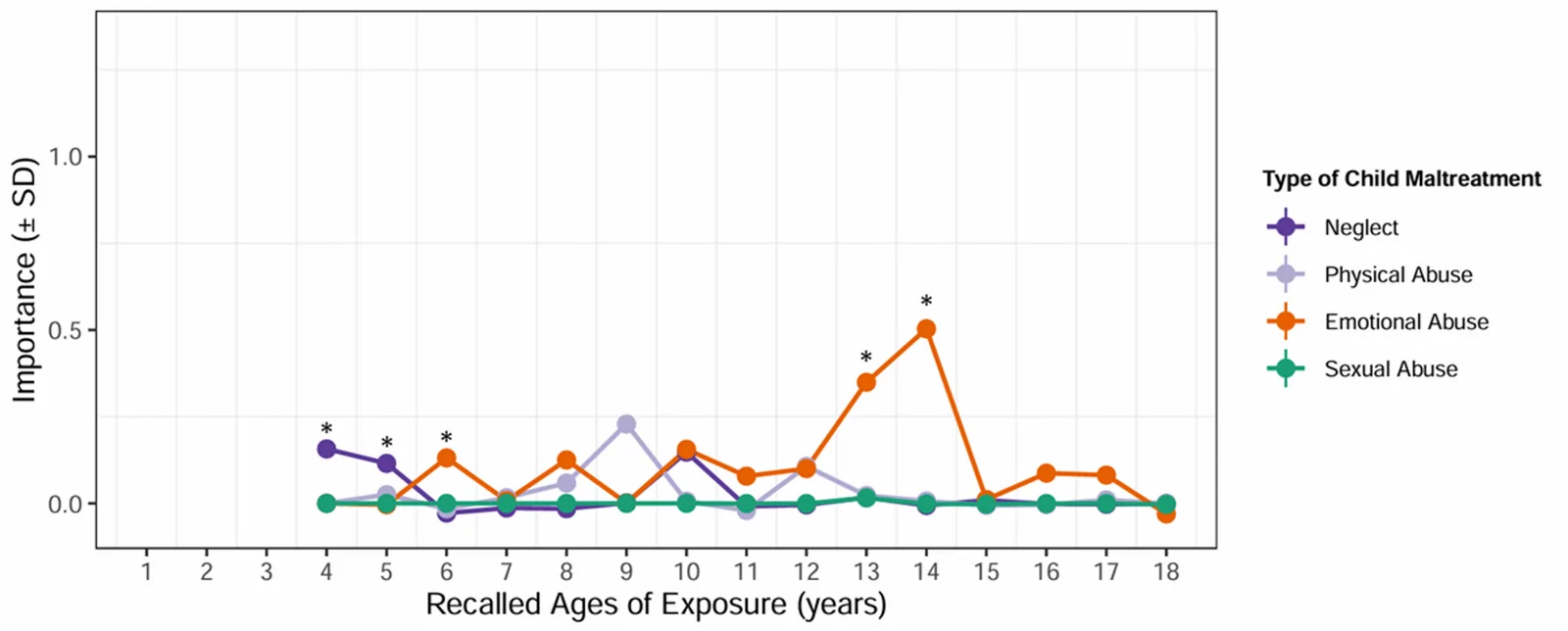
Importance of child maltreatment for adult internalising disorders in men

With regard to IMD in adulthood, especially timing of emotional abuse, i.e. exposure to emotional abuse between ages 10–16 (VI ranging from 0.255 to 1.014) and 18 for women and ages 6 and 13–14 (VI ranging from 0.349 to 0.504) for men was of importance. Besides, for women exposure to neglect at ages 7 and 13, physical abuse at age 16, and sexual abuse at age 11 was of importance of IMD in adulthood, for men exposure to neglect at ages 4 and 5. Results are displayed in Figs. 1 and 2.

Figures 1 and 2: (1) stars display significance importance for outcome based on *p* < 0.05 (two-sided). (2) Model included timing of subtypes CM, multiplicity CM, duration CM, frequency CM, age, and household equivalent income. (3) Valid cases included in models are: Fig. 1*n* = 1264, Fig. 2*n* = 1169. (4) Small values of variable importance (either negative or positive) give the range of unimportance of experienced subtype of CM in this age for internalising disorders in adulthood. (5) Due to minimal memorising capacity in young age, recalled CM at very young age (below the age of three years) was excluded from the analyses. (6) With regard to sexual abuse, the number of persons having experienced sexual abuse in the ages 4 until 10 is relatively small, therefore these results should be interpreted with caution. The same goes for neglect age 18 in women and emotional abuse age 4 in men.

**Fig. 3:**
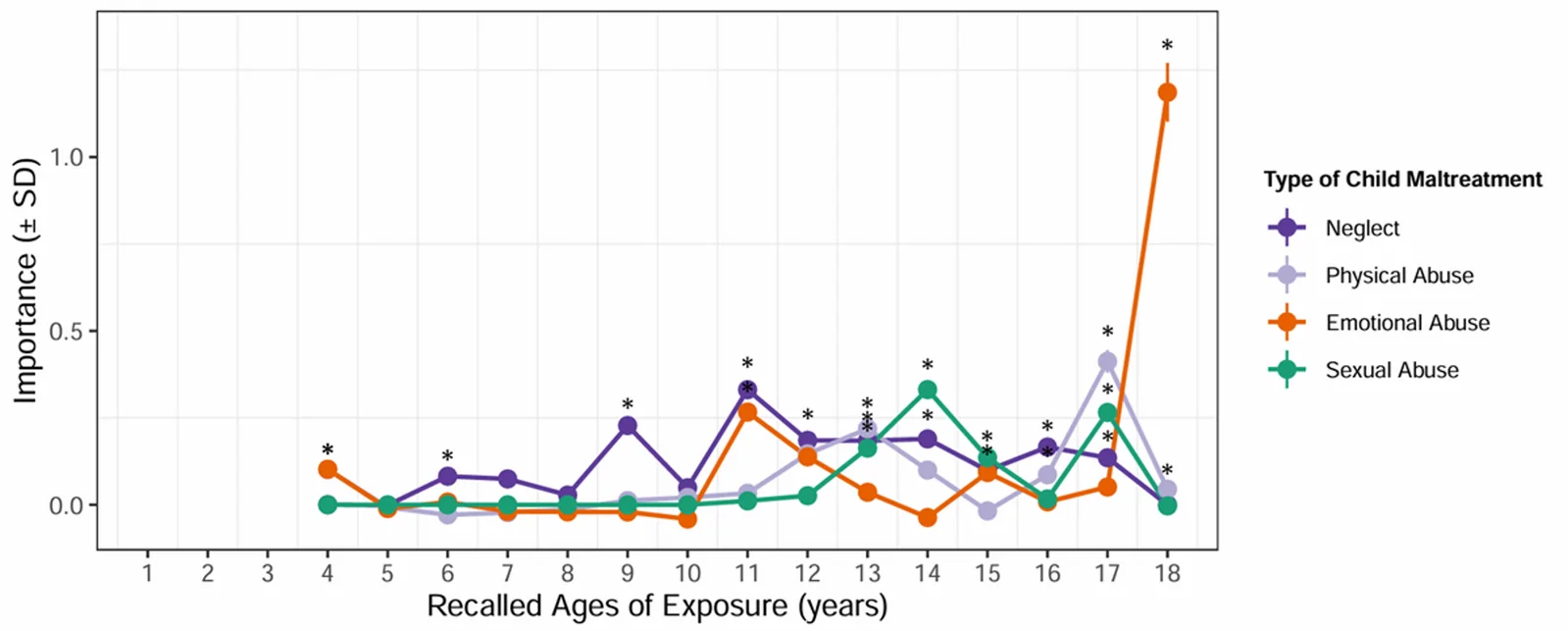
Importance of child maltreatment for adult externalising disorders in women

**Fig. 4:**
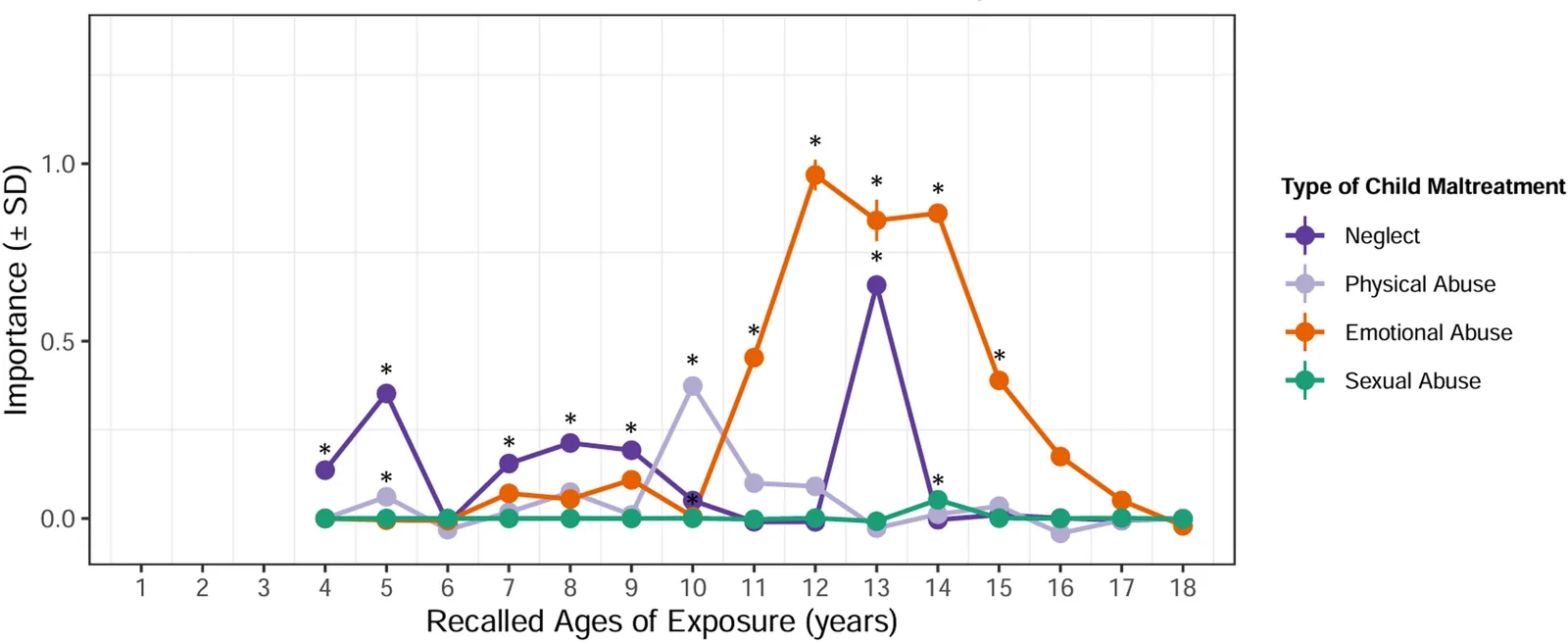
Importance of child maltreatment for adult externalising disorders in men

EMD in adulthood were for women most importantly associated with exposure to sexual abuse at ages 13–15 (VI ranging from 0.136 to 0.331) and 17 and experiences of neglect at ages 6, 9, and 11–17. For men, exposure to emotional abuse at ages 11–16 (VI ranging from 0.174 to 0.968) was most important for EMD in adulthood, followed by exposure to neglect at ages 4 and 5, 7–9, and 13. Besides, for women experiences of physical abuse at ages 4, 13, and 16–18 and experiences of emotional abuse at ages 4, 11, and 18 were of importance for EMD in adulthood. For men this was exposure to physical abuse at ages 5 and 10 and exposure to sexual abuse at ages 10 and 14. Results are displayed in Figs. 3 and 4.

Figures 3 and 4: (1) stars display significance importance for outcome based on *p* < 0.05 (two-sided). (2) Model included timing of subtypes CM, multiplicity CM, duration CM, frequency CM, age, and household equivalent income. (3) Valid cases included in models are: Fig. 3*n* = 1265, Fig. 4*n* = 1173. (4) Small values of variable importance (either negative or positive) give the range of unimportance of experienced subtype of CM in this age for externalising disorders in adulthood. (5) Due to minimal memorising capacity in young age, recalled CM at very young age (below the age of three years) was excluded from the analyses. (6) With regard to sexual abuse, the number of persons having experienced sexual abuse in the ages 4 until 10 is relatively small, therefore these results should be interpreted with caution. The same goes for neglect age 18 in women and emotional abuse age 4 in men.

#### Importance of global scores and covariates

Multiplicity was of importance for adult IMD and EMD in both women and men, especially for IMD in women (VI = 2.365) versus EMD in men (VI = 2.126). Comparative hypothesis testing revealed multiplicity to be more important than timing of CM subtypes except for exposure to emotional abuse at age 18, which was for women more important for EMD (*p* < 0.001). Duration was also of importance for adult IMD and EMD in both women and men, especially for EMD in men (VI = 1.686). For women, emotional abuse at specific ages was more important than duration of CM, i.e., exposure at ages 14 and 15 (*p* < 0.001) for IMD and exposure at age 18 for EMD (*p* < 0.001). Frequency of CM was important for adult IMD and EMD in women, in men only for EMD. Exposure to emotional abuse between the ages 11–15 (*p* < 0.001) for men and at age 17–18 (*p* < 0.001) for women was found to be more important for adult EMD than frequency of CM. For men, this was also found for exposure to neglect at ages 5 and 13 (*p* < 0.001) and for exposure to physical abuse at age 10 (*p* < 0.001). Furthermore, for men exposure to emotional abuse at ages 13 and 14 (*p* < 0.001) was more important than frequency of CM for adult IMD.

With regard to control variables, only household equivalised income was found to be important for EMD in men (negative association). Results are displayed in Supplementary Tables 2a and 2b.

#### Direction and strength of timing of exposure

**Fig. 5:**
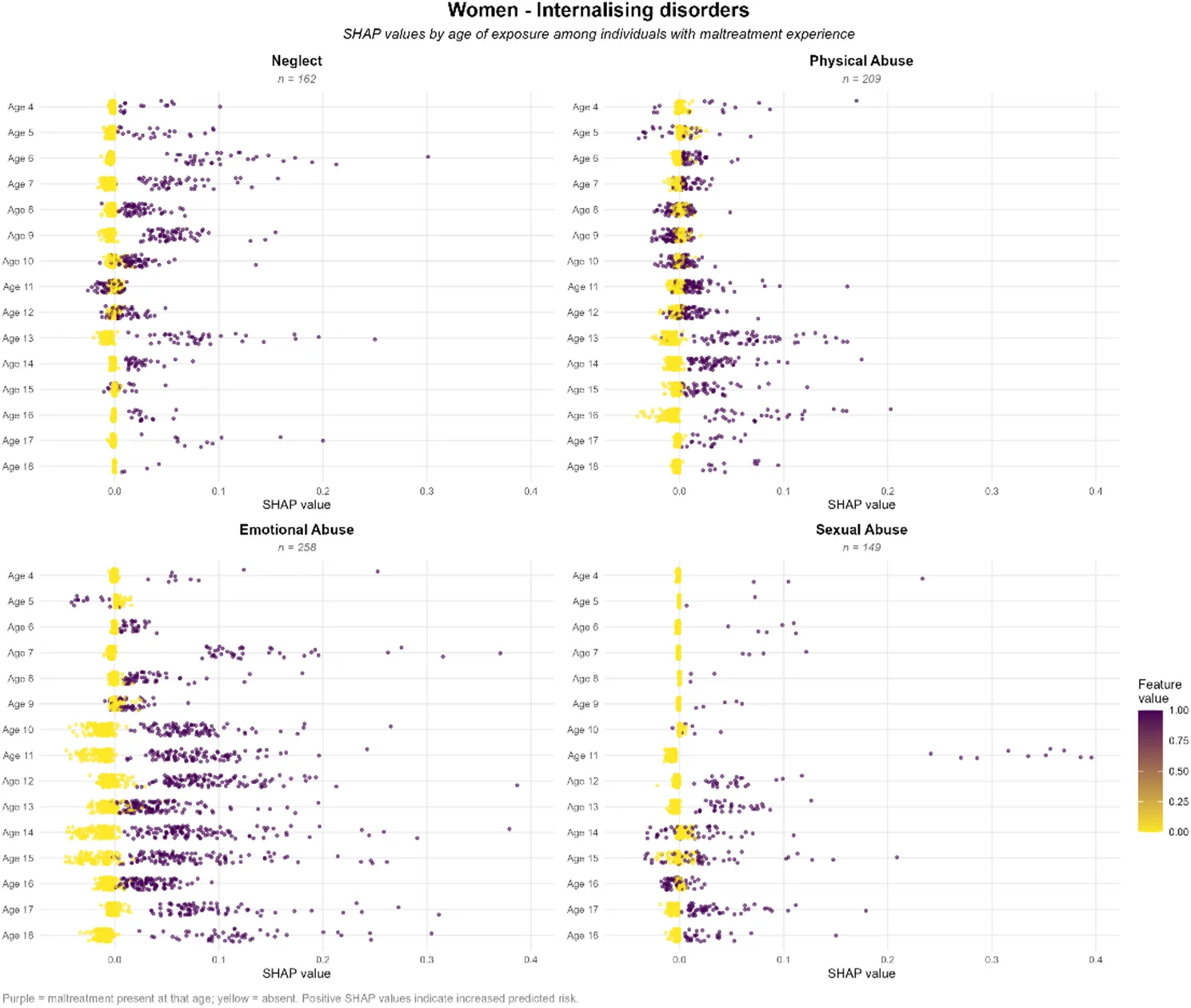
SHAP values by age of child maltreatment subtype exposure for adult internalising disorders in women

**Fig. 6:**
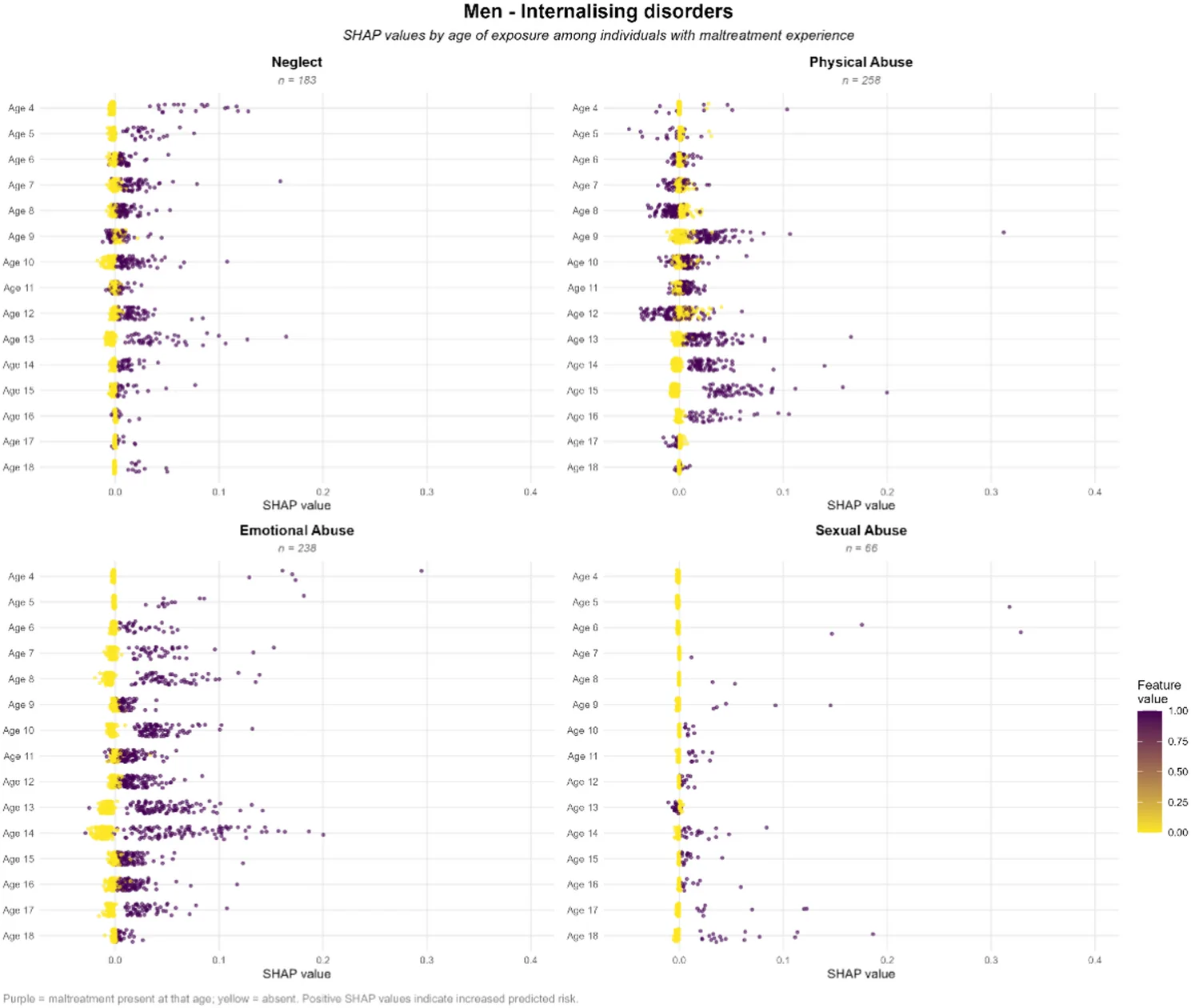
SHAP values by age of child maltreatment subtype exposure for adult internalising disorders in men

**Fig. 7:**
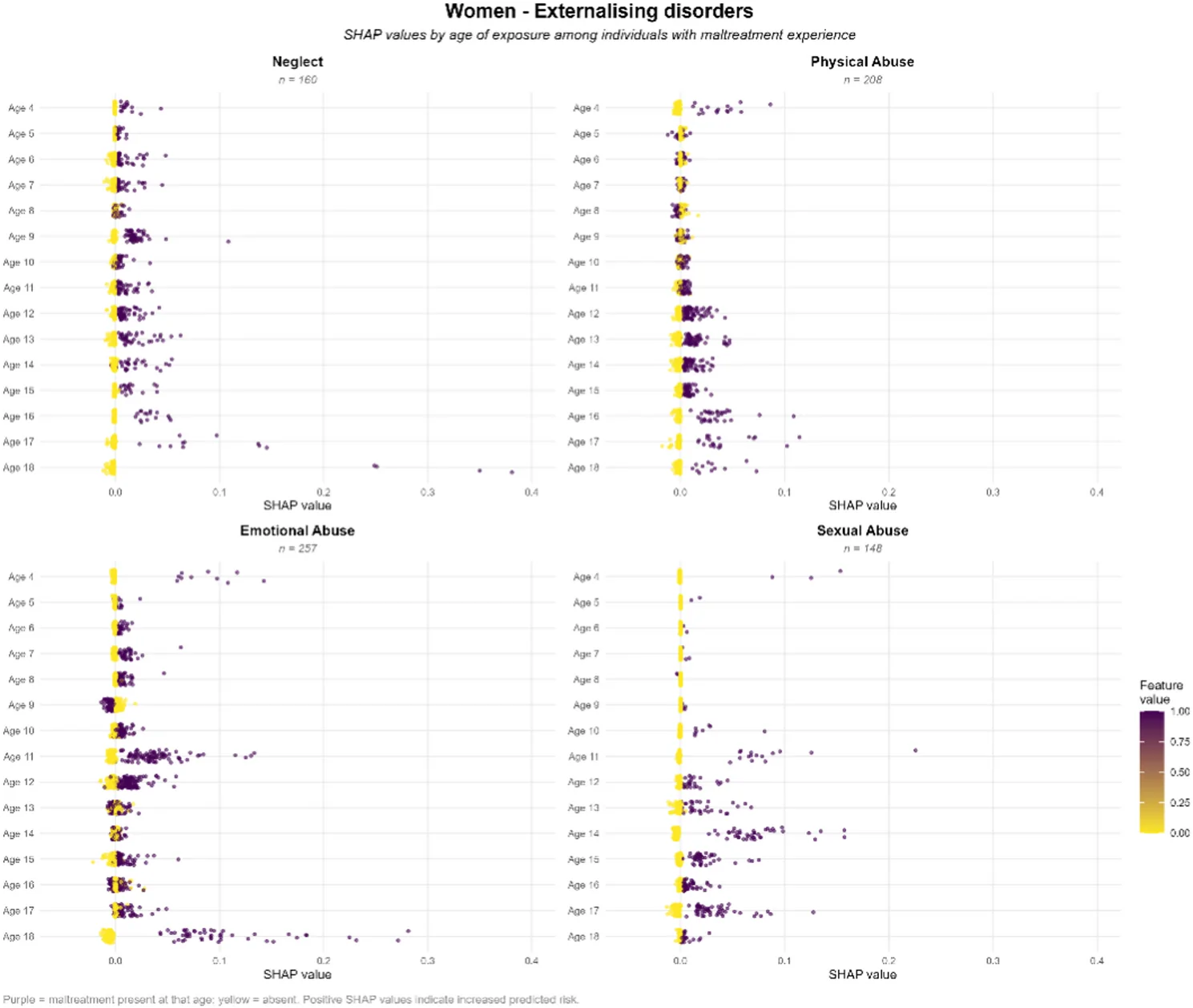
SHAP values by age of child maltreatment subtype exposure for adult externalising disorders in women

**Fig. 8:**
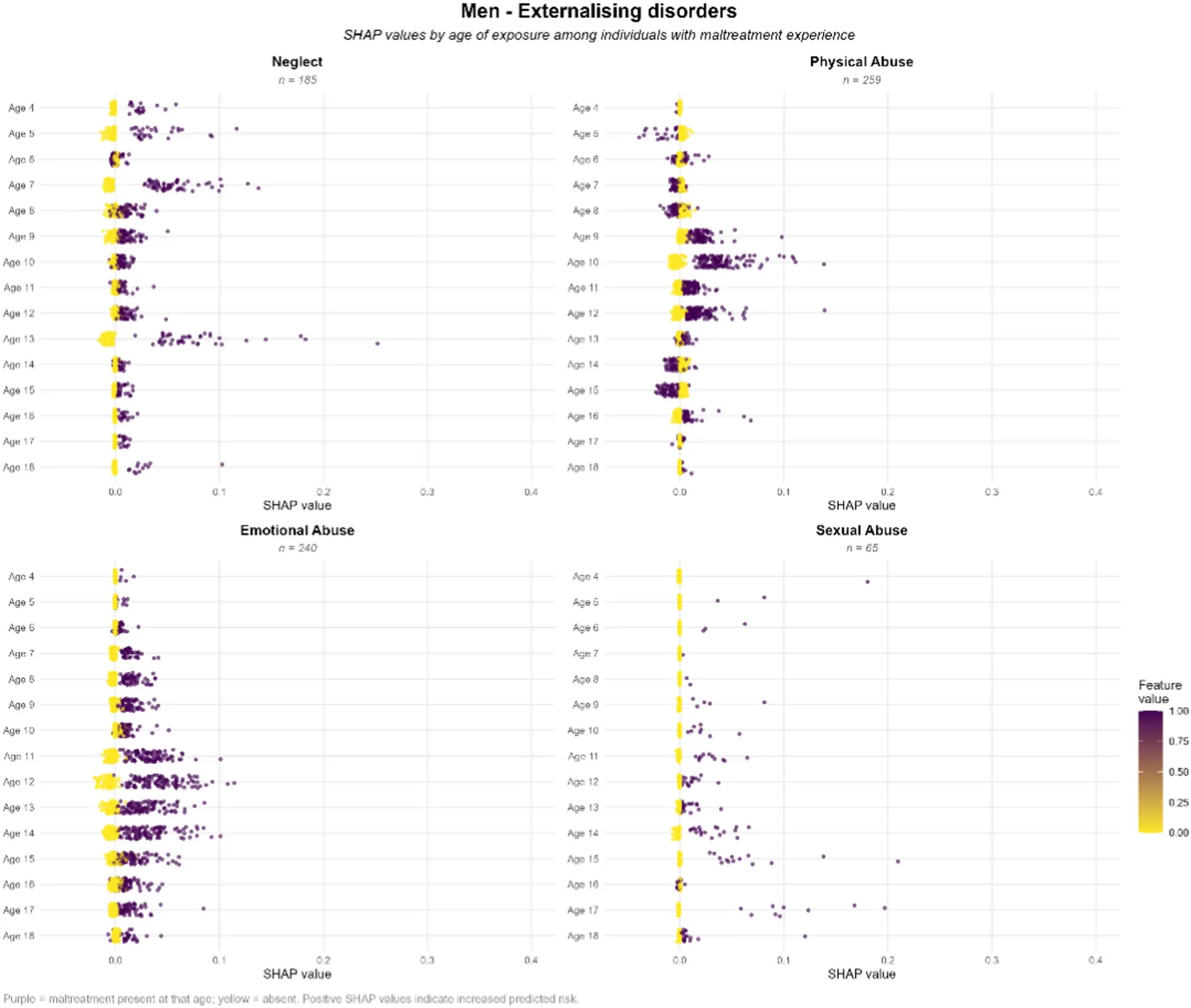
SHAP values by age of child maltreatment subtype exposure for adult externalising disorders in men

The SHAP based beeswarm plots represent individual predictions of timing and subtype of CM exposure for IMD and EMD in women and men. Each point represents one individual, positive SHAP values indicate an increased associated risk for mental disorders in adulthood. Results confirm the findings regarding timing of emotional abuse i.e., exposure to emotional abuse in pre-puberty (over a longer window of years of age for women than men) and its associations with IMD in adulthood. Furthermore, SHAP values confirm the importance of exposure to emotional abuse in puberty for EMD in adulthood in men (Figs. 5–8).

## Discussion

### Interpretation results

Using data from a large population-based survey, this study aimed to identify critical time windows of CM exposure (neglect, emotional, physical, and sexual abuse) that were most strongly associated with internalising and externalising mental disorders in adulthood for women and men and compared their importance to global indicators of CM (multiplicity, duration, and frequency).

Results revealed exposure of emotional abuse in (pre)puberty to be of high importance of IMD in adulthood in both women and men and more important than global CM indicators duration (women) and frequency (men). This finding corresponds with previous studies who found emotional abuse to be of significant importance for dissociative symptoms in adulthood [25]. Additionally, Khan et al. revealed having experienced emotional neglect in childhood to be associated with major depression disorder in adulthood in women [29], whereas Gerke et al., found emotional subtypes of CM experienced in adolescence to be predictive for lifetime major depressive disorder [36]. A review study revealed cumulative effects of CM for depression to be most pronounced in mid-childhood [37]. Furthermore, exposure of emotional abuse in (pre)puberty was of importance of EMD in adulthood in men and more important than overall frequency of CM, which aligns with a study on young adults revealing associations between emotional abuse and externalising mental health problems in men [38]. These findings were confirmed in post-hoc SHAP analyses that also revealed a concentration of positive SHAP values in the period mid-to-late teen years.

Besides of the developmental period for emotional abuse, timing of neglect was important for EMD in adulthood in women (neglect ages 6, 9, and 11–17) and men (neglect ages 4,5, 7–9, and 13). Having been exposed to neglect was associated with lower neurocognitive functions (e.g., language and verbal learning, problem solving) [39]. Shortfall in neurocognitive functions were in turn linked to EMD [40]. With regard to sexual abuse, exposure in puberty (ages 13–15 and 17) and (pre)puberty (ages 10 and 14), respectively for women and men, was important for EMD in adulthood. For physical abuse exposure at very young age, (pre)puberty or late puberty (only women) was of importance for EMD in adulthood. Main changes in the brain occur in early childhood and puberty, these are especially vulnerable phases in which children are more susceptible of negative effects of CM [15].

The highly heterogeneous experiences that are captured with CM as well as its timing have been associated with a broad range of psychopathological [6, 9] and neurobiological outcomes [20, 41]. Association between type and timing of CM and mental disorders in adulthood could be explained by various neurobiological alterations. Previous studies have revealed the important role of the dysregulation of the HPA-axis and autonomic nervous system in stress responses producing physiological changes that are maladaptive in the long term [12]. The age period of the importance of experienced emotional abuse for later mental health as found in this study corresponds to the most sensitive period of the development of the HPA-axis [16] and for major developments in brain structure and function [42]. Besides neurobiological factors, psychological mechanisms might be of importance. CM exposure, especially experiences of emotional abuse, showed a robust negative association with resilience (including emotion regulation and self-efficacy) in adulthood [43]. CM exposure in puberty is associated with emotion regulation difficulties in adulthood, which is a core aspect for psychopathology i.e. its dysregulation is associated with multiple mental disorders [44].

In addition to timing of exposure, our study revealed the importance of multiplicity for IMD and EMD in adulthood in both women and men, which is consistent with a meta-analysis reporting significant increased probabilities for experiencing mental disorders after having experienced multiple subtypes of CM [45]. Furthermore, duration of CM was of importance for adult IMD and EMD in both women and men. This is in line with previous findings showing long-term CM exposure to be associated with higher rates of IMD and EMD [21].

This analysis does not constitute a validated predictive model, nor does it permit causal inference. Permutation importance reflects the strength of association with the outcome after accounting for correlated predictors, rather than a causal effect, and timing-specific causal claims would require an explicit causal identification strategy. What the present approach offers instead is an exploratory, hypothesis-generating ranking of which maltreatment subtype and timing variables carry the greatest relative information about the outcomes in this sample. In this way, it helps narrow a large and correlated variable space and highlights developmental periods that may be particularly worthwhile to examine in future predictive or causal-inference study designs.

### Strengths and limitations

The major advantage of this study is the representativeness of the sample, ensuring a high generalizability of the results as well as the in-depth data on numerous characteristics (i.e., type and timing) of CM collected with a psychometrically strong instrument. cRF analyses enabled us to disentangle the critical windows in timing of subtype of CM for adverse mental health in adulthood.

The major limitation is the retrospective assessment of CM, which validity has been critically debated [46], since little congruence of reports of CM between retrospective and prospective studies was found. Adults might retrospectively overestimate the content of the early childhood memories that survive [47]. However, several studies emphasize that especially events that affect children’s life significantly will remain in memory and can be retained accurately over many years into adulthood [48, 49] herewith endorsing the accurate measurement of child maltreatment experiences using retrospective design. A second problem is that retrospective studies revealed to be more strongly associated with psychopathology [50]. Subjective reports of CM were, however, shown to be highly relevant for adulthood [51]. A recent meta-analysis revealed effects of childhood adversity on psychopathology to be primarily driven by a person’s subjective experience [52]. Besides the remembrance of CM exposure in general, remembering the specific age of experienced events might be problematic. Nonetheless, due to the autobiographical memory an approximate age of experienced events can probably be recalled. Childhood is naturally segmented by e.g., cognitive stages, physical development, school years, which provides a framework for temporal placement of events, i.e., other memories from that age, to help to remember the time point of a specific event [53]. Due to the impaired understanding and remembrance of events happening in early childhood [54, 55], results of recalled CM below the age of four were not shown and interpreted. A recall-bias for neglect presumably exist even longer in early childhood, though this is especially the age at which exposure might have the most serious consequences. Longitudinal assessments of CM in cohorts starting with birth could provide insight into the prevalence of CM in this highly sensitive period. This study only reports on associations and not causality.

## Conclusion

Exposure to CM in (pre)puberty was of high importance of adult mental health; emotional abuse for IMD in women and men, neglect in women versus emotional abuse in men for EMD, and sexual and physical abuse for EMD in both women and men. Also, multiplicity and duration of exposure were of importance for adult mental health. These findings underline the urgence of comprehensive information on CM experiences in both clinical practice and research. Assessment of type and timing of CM exposure could contribute to earlier diagnosing of mental disorders, as well as early intervention to prevent individual suffering and further aggravation of CM experiences with increasing deleterious health outcomes. Despite the protections of the United Nations Convention on the Rights of the Child (UN General Assembly, 1989, Art. 19) CM remains a global challenge.

## Supplementary Information

Below is the link to the electronic supplementary material.

Supplementary Material 1.

## Data Availability

The datasets analysed during the current study as well as the scripts are available from the corresponding author upon reasonable request.

## Abbreviations

CM: Child maltreatment
cRF: Conditioned random forest regression
EMD: Externalising mental disorders
HPA-axis: Hypothalamic-pituitary-adrenal-axis
IMD: Internalising mental disorders
SHAP: SHapley additive explanations
VI: Variable importance
ICD-10: International classification of diseases, 10th revision
