# Supplementary material for: Critical windows: timing of child maltreatment exposure and adult mental health
Source: Child Adolesc Psychiatry Ment Health. 2026 Oct 6;20:139. doi: 10.1186/s13034-026-01188-0 (PMC13647614; doi:10.1186/s13034-026-01188-0)
Supplement: Supplementary file 1 [file 13034_2026_1188_MOESM1_ESM.docx]

**Supplementary Material**

Supplementary Table 1. Descriptive statistics of all internalising and externalising mental disorders stratified for women and men.

|  | Women  (N = 1,278) | Men  (N = 1,188) | Significance (*p*-value) |
| --- | --- | --- | --- |
|  |  |  |  |
| **Internalising disorders** |  |  |  |
| Depression (N, %) |  |  | <0.001 |
| No | 1113, 87.1% | 1096, 92.3% |  |
| Yes | 161, 12.6% | 88, 7.4% |  |
| Anxiety (N, %) |  |  | <0.001 |
| No | 1161, 90.8% | 11741, 96.0% |  |
| Yes | 108, 8.5% | 40, 3.4% |  |
| Burn-out (N, %) |  |  | 0.029 |
| No | 1208, 94.5% | 1141, 96.0% |  |
| Yes | 66, 5.2% | 40, 3.4% |  |
| Posttraumatic stress disorder (N, %) |  |  | 0.444 |
| No | 1250, 97.8% | 1169, 98.4% |  |
| Yes | 25, 2.0% | 18, 1.5% |  |
| Eatig disorders (N, %) |  |  | <0.001 |
| No | 1225, 95.9% | 1173, 98.7% |  |
| Yes | 50, 3.9% | 12, 1.0% |  |
| **Externalising disorders** |  |  |  |
| Personality disorders (N, %) |  |  | 0.074 |
| No | 1241, 97.1% | 1168, 98.3% |  |
| Yes | 30, 2.3% | 16, 1.3% |  |
| Attention deficit disorder (N, %) |  |  | 0.250 |
| No | 1266, 99.1% | 1173, 98.7% |  |
| Yes | 7, 0.5% | 12, 1.0% |  |
| Schizophrenia (N, %) |  |  | 0.357 |
| No | 1275, 99.8% | 1180, 99.3% |  |
| Yes | 1, 0.1% | 3, 0.3% |  |
| Addiction disorders (N, %) |  |  | <0.001 |
| No | 1246, 97.5% | 1100, 92.6% |  |
| Yes | 27, 2.1% | 82, 6.9% |  |

*Notes*: CM = child maltreatment.

Supplementary Table 2a. Importance of child maltreatment characteristics for internalising mental disorders in adulthood in women and men.

|  | Women | | Men | |
| --- | --- | --- | --- | --- |
|  | Imp. | SD | Imp. | SD |
| Timing neglect |  |  |  |  |
| age 4 | 0.002 | 0.000 | **0.157** | 0.007 |
| age 5 | 0.001 | 0.000 | **0.115** | 0.008 |
| age 6 | 0.026 | 0.001 | -0.028 | 0.001 |
| age 7 | **0.145** | 0.005 | -0.013 | 0.001 |
| age 8 | 0.022 | 0.002 | -0.015 | 0.003 |
| age 9 | 0.114 | 0.006 | 0.002 | 0.001 |
| age 10 | 0.034 | 0.003 | 0.148 | 0.007 |
| age 11 | 0.033 | 0.002 | -0.009 | 0.001 |
| age 12 | 0.063 | 0.003 | -0.005 | 0.001 |
| age 13 | **0.133** | 0.005 | 0.017 | 0.002 |
| age 14 | -0.01 | 0.001 | -0.006 | 0.001 |
| age 15 | -0.003 | 0.000 | 0.009 | 0.001 |
| age 16 | -0.004 | 0.000 | -0.002 | 0.000 |
| age 17 | -0.001 | 0.000 | -0.003 | 0.000 |
| age 18 | 0.000 | 0.000 | 0.000 | 0.000 |
| Timing physical abuse |  |  |  |  |
| age 4 | -0.001 | 0.000 | 0.000 | 0.000 |
| age 5 | 0.020 | 0.001 | 0.025 | 0.001 |
| age 6 | -0.012 | 0.002 | -0.015 | 0.001 |
| age 7 | 0.011 | 0.002 | 0.016 | 0.003 |
| age 8 | -0.014 | 0.002 | 0.059 | 0.005 |
| age 9 | 0.021 | 0.003 | 0.229 | 0.012 |
| age 10 | 0.003 | 0.003 | 0.006 | 0.003 |
| age 11 | -0.020 | 0.002 | -0.02 | 0.002 |
| age 12 | -0.012 | 0.005 | 0.107 | 0.003 |
| age 13 | 0.070 | 0.008 | 0.023 | 0.004 |
| age 14 | 0.001 | 0.003 | 0.007 | 0.003 |
| age 15 | -0.009 | 0.003 | -0.006 | 0.002 |
| age 16 | **0.190** | 0.008 | -0.004 | 0.001 |
| age 17 | 0.009 | 0.001 | 0.010 | 0.000 |
| age 18 | 0.008 | 0.001 | 0.000 | 0.000 |
| Timing emotional abuse |  |  |  |  |
| age 4 | 0.008 | 0.000 | 0.000 | 0.000 |
| age 5 | -0.010 | 0.001 | -0.004 | 0.000 |
| age 6 | 0.049 | 0.004 | **0.131** | 0.007 |
| age 7 | 0.025 | 0.003 | 0.008 | 0.005 |
| age 8 | 0.054 | 0.004 | 0.125 | 0.008 |
| age 9 | 0.010 | 0.004 | 0.001 | 0.003 |
| age 10 | **0.486** | 0.024 | 0.156 | 0.009 |
| age 11 | **0.429** | 0.024 | 0.079 | 0.005 |
| age 12 | **0.287** | 0.015 | 0.100 | 0.008 |
| age 13 | **0.465** | 0.023 | **0.349** | 0.021 |
| age 14 | **1.014** | 0.030 | **0.504** | 0.027 |
| age 15 | **0.693** | 0.022 | 0.011 | 0.006 |
| age 16 | **0.255** | 0.009 | 0.087 | 0.006 |
| age 17 | 0.145 | 0.006 | 0.082 | 0.007 |
| age 18 | **0.223** | 0.009 | -0.030 | 0.001 |
| Timing sexual abuse |  |  |  |  |
| age 4 | 0.000 | 0.000 | 0.000 | 0.000 |
| age 5 | 0.000 | 0.000 | 0.000 | 0.000 |
| age 6 | 0.000 | 0.000 | 0.000 | 0.000 |
| age 7 | 0.000 | 0.000 | 0.000 | 0.000 |
| age 8 | 0.000 | 0.000 | 0.000 | 0.000 |
| age 9 | 0.000 | 0.000 | 0.000 | 0.000 |
| age 10 | 0.000 | 0.000 | 0.000 | 0.000 |
| age 11 | **0.171** | 0.006 | -0.001 | 0.000 |
| age 12 | 0.034 | 0.001 | -0.001 | 0.000 |
| age 13 | 0.039 | 0.001 | 0.016 | 0.000 |
| age 14 | 0.011 | 0.001 | -0.001 | 0.000 |
| age 15 | 0.011 | 0.002 | -0.002 | 0.000 |
| age 16 | -0.017 | 0.001 | 0.000 | 0.000 |
| age 17 | 0.028 | 0.003 | 0.000 | 0.000 |
| age 18 | -0.002 | 0.000 | -0.003 | 0.000 |
| Global scores CM |  |  |  |  |
| Multiplicity | **2.365** | 0.125 | **1.217** | 0.057 |
| Duration | **0.647** | 0.034 | **0.511** | 0.034 |
| Frequency | **1.495** | 0.060 | 0.324 | 0.0233 |
| Covariates |  |  |  |  |
| Age at survey participation | 0.127 | 0.005 | 0.266 | 0.0011 |
| Migration background | -0.021 | 0.003 | 0.302 | 0.012 |
| Partnership (living with partner) | 0.206 | 0.011 | 0.257 | 0.008 |
| Net household equivalised income | 0.082 | 0.005 | 0.149 | 0.008 |

*Notes*: Imp. = Importance, CM = child maltreatment, valid cases model women: n=1264, valid cases model men: n=1169, bold values are significant (p<0∙05). Regarding sexual abuse, the number of persons having experienced sexual abuse in the ages 4 until 10 is relatively small, therefore these results should be interpreted with caution. The same goes for neglect age 18 in women and emotional abuse age 4 in men.

Supplementary Table 2b. Importance of child maltreatment characteristics for externalising mental disorders in adulthood in women and men.

|  | Women | | Men | |
| --- | --- | --- | --- | --- |
|  | Imp. | SD | Imp. | SD |
| Timing neglect |  |  |  |  |
| age 4 | 0.000 | 0.000 | **0.136** | 0.006 |
| age 5 | -0.001 | 0.000 | **0.352** | 0.015 |
| age 6 | **0.082** | 0.006 | -0.014 | 0.003 |
| age 7 | 0.074 | 0.005 | **0.155** | 0.009 |
| age 8 | 0.028 | 0.004 | **0.213** | 0.017 |
| age 9 | **0.228** | 0.010 | **0.192** | 0.011 |
| age 10 | 0.049 | 0.005 | 0.050 | 0.01 |
| age 11 | **0.331** | 0.024 | -0.009 | 0.003 |
| age 12 | **0.185** | 0.021 | -0.010 | 0.004 |
| age 13 | **0.185** | 0.015 | **0.658** | 0.027 |
| age 14 | **0.189** | 0.015 | -0.004 | 0.001 |
| age 15 | **0.098** | 0.005 | 0.01 | 0.002 |
| age 16 | **0.166** | 0.008 | 0.001 | 0.001 |
| age 17 | **0.135** | 0.009 | -0.006 | 0.000 |
| age 18 | 0.000 | 0.000 | 0.000 | 0.000 |
| Timing physical abuse |  |  |  |  |
| age 4 | **0.101** | 0.004 | 0.000 | 0.000 |
| age 5 | -0.007 | 0.001 | **0.061** | 0.002 |
| age 6 | -0.029 | 0.002 | -0.032 | 0.001 |
| age 7 | -0.023 | 0.002 | 0.017 | 0.002 |
| age 8 | -0.013 | 0.002 | 0.074 | 0.006 |
| age 9 | 0.012 | 0.002 | 0.010 | 0.003 |
| age 10 | 0.021 | 0.005 | **0.373** | 0.020 |
| age 11 | 0.033 | 0.009 | 0.100 | 0.009 |
| age 12 | 0.146 | 0.016 | 0.090 | 0.007 |
| age 13 | **0.219** | 0.022 | -0.027 | 0.004 |
| age 14 | 0.100 | 0.012 | 0.012 | 0.002 |
| age 15 | -0.018 | 0.004 | 0.035 | 0.005 |
| age 16 | **0.086** | 0.012 | -0.042 | 0.002 |
| age 17 | **0.412** | 0.033 | -0.005 | 0.000 |
| age 18 | **0.045** | 0.003 | 0.000 | 0.000 |
| Timing emotional abuse |  |  |  |  |
| age 4 | **0.102** | 0.004 | 0.000 | 0.000 |
| age 5 | -0.011 | 0.002 | -0.005 | 0.000 |
| age 6 | 0.008 | 0.002 | -0.006 | 0.001 |
| age 7 | -0.019 | 0.001 | 0.0710 | 0.005 |
| age 8 | -0.020 | 0.002 | 0.055 | 0.007 |
| age 9 | -0.021 | 0.004 | 0.109 | 0.010 |
| age 10 | -0.041 | 0.006 | 0.005 | 0.005 |
| age 11 | **0.267** | 0.019 | **0.453** | 0.024 |
| age 12 | 0.138 | 0.021 | **0.968** | 0.043 |
| age 13 | 0.036 | 0.015 | **0.840** | 0.058 |
| age 14 | -0.037 | 0.006 | **0.860** | 0.016 |
| age 15 | 0.093 | 0.014 | **0.389** | 0.018 |
| age 16 | 0.009 | 0.004 | **0.174** | 0.006 |
| age 17 | 0.051 | 0.007 | 0.051 | 0.004 |
| age 18 | **1.186** | 0.084 | -0.021 | 0.002 |
| Timing sexual abuse |  |  |  |  |
| age 4 | 0.000 | 0.000 | 0.000 | 0.000 |
| age 5 | 0.000 | 0.000 | 0.000 | 0.000 |
| age 6 | 0.000 | 0.000 | 0.000 | 0.000 |
| age 7 | 0.000 | 0.000 | 0.000 | 0.000 |
| age 8 | 0.000 | 0.000 | 0.000 | 0.000 |
| age 9 | 0.000 | 0.000 | 0.000 | 0.000 |
| age 10 | 0.000 | 0.000 | **0.000** | 0.000 |
| age 11 | 0.011 | 0.000 | -0.002 | 0.001 |
| age 12 | 0.026 | 0.003 | 0.001 | 0.000 |
| age 13 | **0.163** | 0.009 | -0.008 | 0.000 |
| age 14 | **0.331** | 0.018 | **0.053** | 0.002 |
| age 15 | **0.136** | 0.010 | 0.001 | 0.000 |
| age 16 | 0.017 | 0.003 | 0.000 | 0.000 |
| age 17 | **0.266** | 0.014 | 0.001 | 0.000 |
| age 18 | -0.003 | 0.000 | -0.002 | 0.000 |
| Global scores CM |  |  |  |  |
| Multiplicity | **1.035** | 0.070 | **2.126** | 0.071 |
| Duration | **0.806** | 0.060 | **1.686** | 0.080 |
| Frequency | **0.969** | 0.045 | **0.320** | 0.023 |
| Covariates |  |  |  |  |
| Age at survey participation | 0.243 | 0.021 | 0.0202 | 0.005 |
| Migration background | -0.041 | 0.003 | 0.089 | 0.006 |
| Partnership (living with partner) | 0.025 | 0.011 | 0.100 | 0.006 |
| Net household equivalised income | 0.080 | 0.007 | **0.931** | 0.031 |
|  |  |  |  |  |

*Notes*: Imp. = Importance, CM = child maltreatment, valid cases model women: n=1265, valid cases model men: n=1173, bold values are significant (p<0.05). Regarding sexual abuse, the number of persons having experienced sexual abuse in the ages 3 until 10 is relatively small, therefore these results should be interpreted with caution. The same goes for neglect age 17-18 in women and emotional abuse age 3-4 in men.
